# Systematic Screening for Venous Thromboembolic Events in COVID-19 Pneumonia

**DOI:** 10.1055/s-0040-1713167

**Published:** 2020-06-08

**Authors:** Gaël Grandmaison, Antoine Andrey, Daniel Périard, Rolf P. Engelberger, Guillaume Carrel, Sébastien Doll, Jean-Baptiste Dexpert, Caroline Krieger, Hatem Ksouri, Daniel Hayoz, Govind Sridharan

**Affiliations:** 1 Internal Medicine, HFR Hôpital Cantonal, Fribourg, Switzerland; 2 Intensive Care Unit, HFR Hôpital Cantonal, Fribourg, Switzerland; 3 Angiology Unit, HFR Hôpital Cantonal, Fribourg, Switzerland

The new coronavirus disease 2019 (COVID-19) pandemic spreads worldwide, provoking pneumonia, acute respiratory distress syndrome (ARDS) and death. Recently, the medical community has been alerted by reports of coagulation disorders, 1 2 with arterial and venous thromboembolic events (VTEs) among patients with acute COVID-19. 3 4 5 6 7 8 9 This disproportionate incidence of vascular events seems to be linked to a strong inflammatory response against the severe acute respiratory syndrome coronavirus 2 (SARS-CoV-2), and to an infection of endothelial cells, with endotheliitis, viral inclusions and endothelial cells apoptosis. 10 11 This systemic endothelial injury, associated with the host inflammatory response against SARS-CoV-2, results in activation of coagulation with arterial, venous, catheter and dialysis filter thromboses. Despite several reports, the incidence of VTEs is not precisely known. As VTE is a treatable condition that may adversely affect the survival of already severely compromised patients, we performed a single cross-sectional systematic search for VTEs among the COVID-19 patients in the intensive care unit (ICU) and medicine ward.

All patients hospitalized in our institution (a 320 beds university hospital) with ARDS or pneumonia, and a SARS-CoV-2 positive PCR test, were screened for VTEs on the 7th April 2020. Vascular specialists inspected and performed a complete duplex ultrasound of the neck, of the upper and of the lower limb veins. In case of inconclusive calf veins examination, the latter was repeated within 7 days. Thoracic computed tomography angiography (angio-CT) was performed in case of clinical suspicion of pulmonary embolism (PE) while the duplex ultrasound was negative. Fifty eight patients, 29 in the ICU and 29 in the medicine ward, were screened by duplex ultrasound; 8 of 58 (13.8%) required a second duplex ultrasound examination of leg veins within 7 days and 16 of 58 (27.5%) had a thoracic angio-CT performed.

In the ICU, VTEs were found in 17 (58.6%) of the 29 patients ( Table 1 ), affecting all investigated sites. Although deep venous thromboses (DVTs) of the calf veins were the most frequent findings (15 patients), internal jugular DVTs and proximal limb DVTs were also found in 5 patients. Two patients had PEs (1 intermediate-high risk, and 1 low risk PE). Multi-site VTEs were found in 11 patients. In the ICU, VTEs were found after 6 (1 to 15) days of ventilation (14 mechanical and 3 non-invasive ventilation), with little or no clinical suspicion. Most VTEs occurred despite thromboprophylaxis with standard dose of low molecular weight or unfractionated heparins. The most significant laboratory findings were high D-dimer levels in VTE-patients, compared with non VTE-patients at time of screening ( Table 1 ).

**Table 1:** Clinical presentation of VTE among the 29 patients with COVID-19 ARDS in the ICU

	VTE (17 patients)	No VTE (12 patients)	*p* -value
Age (y)	66 (37–79)	65 (46–79)	NS
Male	64.7% (11)	58.3% (7)	NS
**VTE localization**			
PE	11.8% (2)		
Proximal lower limb DVT	17.6% (3)		
Distal lower limb DVT	88.2% (15)		
Upper limb/neck DVT	11.8% (2)		
Bilateral/multisite	64.7% (11)		
Catheter-related	23.5% (4)		
BMI	27 (20 to 34)	28 (19 to 33)	NS
Active cancer	5.9% (1)	8.3% (1)	NS
Previous thrombotic event	5.9% (1)	8.3% (1)	NS
**Duration up to VTE screening (d)**			
COVID symptoms	18 (11–36)	19 (8–28)	NS
Mechanical ventilation/NIV	6 (1–15)	5 (1–16)	NS
SOFA score	7 (2–14)	8 (3–15)	NS
PaO2/FiO2 ratio	96 (59–152)	107 (77–239)	NS
D-dimer μg/L	8760 (1300–32000)	3150 (3100–16100)	<0.01
LDH U/L	867 (498–1785)	695 (519–1180)	<0.05
C-reactive protein mg/L	306 (20–615)	242 (170–418)	NS
Fibrinogen g/L	6.3 (1.6–8.0)	6.0 (4.0–9.0)	NS
Platelet count 10 ^9^ /L	321 (205–522)	359 (48–717)	NS
Ferritin µg/L	1228 (642–5355)	1834 (255–3812)	NS
**Anticoagulation at VTE diagnosis**			
None	11.8% (2) a	0	NS
Prophylactic	82.3% (14)	100%(12)	NS
Therapeutic	5.9% (1)	0	NS

Abbreviations: BMI, body mass index; DVT, deep venous thrombosis; ICU, intensive care unit; NIV, noninvasive ventilation; PE, pulmonary embolism; SOFA, sequential organ failure assessment score; VTE, venous thromboembolic event.

Note: Values are expressed in proportion (and absolute number) and in median and range. Comparison by Wilcoxon ranksum test.

a VTE were diagnosed at ICU admission simultaneously to COVID infection.

Among the 29 patients hospitalized on the medicine ward who did not require ICU or mechanical ventilation, VTEs were found in 6 (20.7%) patients: distal DVTs in 6 patients, proximal DTVs in 2 patients, PEs with low risk in 2 patients, multisite VTEs in 4 patients. One patient with multisite VTEs had also an acute arterial thrombosis of the iliac artery, with distal embolization in the foot arch. These events occurred despite anticoagulation for 22 of 29 patients (75.8%) at prophylactic (55.2%) or even therapeutic dose (20.7%).

This survey shows that COVID-19 patients requiring mechanical or non-invasive ventilation are at very high risk of multiple VTEs, despite conventional thromboprophylaxis routinely administered at time of our survey (subcutaneous enoxaprin 40 mg/day or unfractioned heparin 5000 Units twice a day). Applying systematic VTE screening in these patients seems to reveal a much higher incidence than previously reported in observational studies. In line with our report, Llitjos and colleagues found an overall VTE incidence of 69% in 2 French ICU, where duplex scan ultrasound is performed as a standard of care. 12 In their multi-centric retrospective study of 388 COVID-19 patients, Lodigiani and colleagues found a VTE incidence of 27.6% in the ICU, and 6.6% in the general ward, where VTEs were not systematically searched for. 13 This difference calls for systematic screening by duplex ultrasound, at regular intervals, for COVID-19 patients. 14 15 Interestingly, VTEs may affect ambulatory patients or be the reason for hospitalization, since a significant proportion of VTEs were diagnosed within 24 hours of hospital admission in our survey and in the study by Lodigiani and colleagues. 13

As consequence of our findings, we have adopted the following measures with promising results. In ICU patients, confirmed VTEs were mainly treated either with subcutaneous enoxaparin 1mg/kg twice a day or iv continuous unfractioned heparin; and medical ward patients were treated with rivaroxaban at therapeutic doses. Patients were followed by clinical evaluation and repeated duplex ultrasound of the index venous thrombosis. Under therapeutic anticoagulation, we did not observe any extension of thrombosis or new symptomatic event. For those without VTE, the thromboprophylaxis was reinforced to enoxaparin 40 mg twice a day (or 60mg twice a day for> 120 kg) in the ICU and enoxaparin 40 mg once a day (or 60mg once a day for> 80 kg) in the medicine ward. During the 4 weeks following the introduction of this new regimen, we observed a dramatic but not complete reduction of new VTE. In conclusion, based on our survey, we believe that VTE should be prevented by reinforced drug thromboprophylaxis. In parallel, intermittent (in the ICU) or permanent (medicine ward) leg compression could be useful if available and not contra-indicated. VTEs should be searched by regular clinical evaluation and imaging, since the reduction of these events may decrease the morbidity of COVID-19 pneumonia, especially in the ICU. In our experience, a routine four limbs and neck compression duplex ultrasound was able to diagnose a large number of otherwise missed VTE without harm to the patients.
